# Knowledge, attitude and practices of HIV post exposure prophylaxis amongst health workers in Lagos University Teaching Hospital

**DOI:** 10.11604/pamj.2014.19.172.4718

**Published:** 2014-10-20

**Authors:** Sarah Ajibola, Akinsegun Akinbami, Charles Elikwu, Majeed Odesanya, Ebele Uche

**Affiliations:** 1 Department of Haematology Ben Carson School of Medicine, Babcock University Teaching Hospital, Ilisan-Remo, Ogun State, Nigeria; 2 Department of Haematology, Lagos State University College of Medicine, Ikeja, Lagos, Nigeria; 3 Department of Medical Microbiology Ben Carson School of Medicine, Babcock University Teaching Hospital, Ilisan-Remo, Ogun State, Nigerian; 4 Oak Hospitals Ikorodu, Lagos, Nigeria

**Keywords:** Post exposure prophylaxis, human immunodeficiency virus, Health care workers

## Abstract

**Introduction:**

Timely PEP after needle stick exposure to high risk body fluids can reduce the rate of occupational transmission significantly. Ignorance of this may increase the risk of seroconversion to HIV for healthcare workers. This study was conducted with the aim of demonstrating the current level of knowledge and practise of healthcare workers as regards PEP.

**Methods:**

This was a cross-sectional study, pretested questionnaire were self administered to 372 health workers from various clinical specialties. The responses were collated and analyzed; results were presented in frequency tables.

**Results:**

This study revealed a high level of awareness among the respondents as 83.3% were aware of PEP. Despite the high level of awareness, respondents still have an inadequate knowledge about PEP, only 32% of the respondents could name at least two of the recommended drugs for PEP, only 54.0% of respondents knew when to commence PEP following occupational exposure to HIV. There was a low level of practice of PEP among the respondents as only 6.3% of respondents had PEP despite occurrence of needle stick injury.

**Conclusion:**

This study revealed a general low level use of PEP despite the average knowledge of PEP and the favourable attitude towards HIV PEP amongst the respondents.

## Introduction

The HIV/AIDS pandemic marks a severe developmental crisis in Africa which remains by far the most affected region in the world. With the very high prevalence of sharps injuries, low rate of reporting and use of Post exposure prophylaxis (PEP), the expected national incidence may be seriously underestimated. Prevention of exposure remains the most effective measure to reduce the risk of HIV transmission to health workers but timely PEP, after needle stick exposure to high risk body fluids can reduce the rate of occupational transmission significantly. Guidance on how to deal with incidents of exposure to potentially infectious material has been recommended by Centers for Disease Control of U.S.A. for those workers thought to be exposed to blood borne viruses especially HIV which causes the highest level of anxiety amongst health workers [1]. Percutaneous injury, usually inflicted by a hollow-bore needle, is the most common mechanism of occupational HIV transmission. The CDC estimates that more than 380,000 needle stick injuries occur in hospitals each year; approximately 61% of these injuries are caused by hollow bore devices [2]. The World Health Report 2002 estimates that 2.5% of HIV cases among health care workers worldwide are the result of occupational exposure [3]. Most people at risk of occupational exposures are in developing countries where there is a paucity of standard reporting protocols [3].

The distribution of exposures to blood borne pathogens among different cadre of health care workers (HCWs) is an important variable in implementing preventive measure. Some studies have indicated that exposures to blood-borne infections in healthcare settings are most frequent with nurses than other HCWs. In a study carried out among health care workers in England, Wales and Northern Ireland between July 1997 and June 2000, it was discovered that midwives were the most common group exposed to blood borne viruses followed by doctors [4]. A similar study in Brazil found the same distributions of exposures [5]. In most studies percutaneous exposure is the most common route of exposure. Post exposure prophylaxis (PEP) is just what the name suggests; PEP is any prophylactic (preventive) treatment started immediately after exposure to a pathogen (such as a disease causing virus) in order to prevent infection by the pathogen and the development of diseases [1]. In the case of HIV infection, PEP is short term antiretroviral treatment given to reduce the likelihood of HIV infection after potential exposure either occupationally or through sexual intercourse. Within the health sector, PEP should be provided as part of a comprehensive universal precaution package that reduces staff exposure to infectious hazards at work. The introduction of an occupational exposure program has many benefits, including optimal management of injuries and acquisition of data on infection control measures and may protect health care institutions from false claims for compensation [6].

Providing relevant information on PEP for the health care professionals would help to prevent the transmission of HIV, provide epidemiological data, identify unsafe practices, and reduce anxiety, and/or increase staff retention and productivity. However studies have shown that there is an information gap in the health care setups. For instance a study done in Governmental Health Institutions in Jimma zone and Jimma City in Ethopia in 2008 indicated 81.6% of HCWS exposed did not use post-exposure prophylaxis [7]. A national study in Kenya also showed, among those who were knowledgeable, only 45% sought HIV PEP. The main reasons for not seeking PEP among this group was lack of sufficient information (35%) followed by fear of the process and what could follow (28%) [8]. This research study was conducted primarily to determine the current level of knowledge, assess changing attitudes to, and determine the level of practice of PEP among medical and nursing personnel using Lagos University Teaching Hospital (LUTH) as a case study.

## Methods

The study is a descriptive study conducted in the clinical departments at the Lagos University Teaching Hospital (LUTH). LUTH is a tertiary referral hospital and training centre for both undergraduate and postgraduate doctors and nurses in various specialties and a research institute in South-West Nigeria. It has over a thousand HCWs, the bulk of which comprises of nurses and resident doctors undergoing postgraduate training.

### Study population

The study population included medical and nursing personnel working in clinical departments with the possibility of occupational exposure to blood borne viruses. Other supporting healthcare personnel in the hospital were excluded.

### Data collection

A self designed, structured questionnaire having the common Sociodemographic characteristics and questions that can assess the levels of their knowledge, attitude and practice towards PEP for HIV was prepared by the research team. A pre-test using the questionnaire was conducted among fifteen percent of the total sample size that is not to be included in the study. Questions were modified accordingly after the pre-test had been conducted to elicit the desired results. Pretested questionnaires were self administered to 372 HCWs from various clinical sub specialties. In this study, adequate knowledge was assessed by knowing what PEP is, when to commence HIV PEP after occupational exposure and duration of use of drugs recommended for HIV PEP. Attitude was assessed by whether or not the respondent had a positive attitude towards HIV PEP. Practice was assessed by the actual usage and completion of duration of use of drugs recommended for HIV PEP after occupational exposure.

Eight questions were prepared to assess the knowledge of respondents about PEP for HIV and those respondents who scored greater than or equal to 70% were considered to have adequate knowledge, knowledge is considered inadequate when the correct answer of respondents is < 70% of the eight knowledge questions. A seven item question was used to assess participants’ attitude towards PEP for HIV and those who score 70% and above were considered as having good attitude. To assess the practice of respondents’ seven questions were prepared and those who answered “Yes” to more than 70% of the questions were considered as if they are practicing PEP for HIV.

### Data analysis

Data was analysed using the Epi-Info Statistical Package -Version 16. The results were presented in frequency tables.

## Results

Out of the 372 questionnaires distributed, 300 questionnaires were returned giving a response rate of 80.7%. The mean age of the respondents was 36.81± 15.8 years. The overall female: male ratio was 2:1. 158(52.7%) were single, 125(41.7%) were married while 17(5.7%) were widowed. Majority (52%) of the respondents were doctors in various specialities (Table 1). One respondent was HIV positive, 74.0% were HIV negative while 25.7% did not know their HIV status. The majority of the respondents 83.3% expressed good knowledge of HIV PEP and what it meant. Most respondents who are aware of PEP are aware that PEP reduces the transmission of HIV following exposure (Table 2). On knowledge on the recommended drugs for HIV-PEP only118 (39.3%) of the respondents could not name any of the drugs, majority 182 (60.7%) of the respondents could name at least one of the recommended drugs for PEP. Less than half of the respondents 46(15.3%) knew the correct duration for the use of HIV PEP.

**Table 1:** Socio-demographic characteristics of respondents’

Variables		N (%)
Age of respondents	20-30 years	177(59)
	31-40 years	63(21)
	>40 years	60(20)
Sex	Male	91(30.3)
	Female	209(69.7)
Marital status	Married	125(41.7)
	Single	158(52.7)
	Widowed	17(5.7)
Religion	Christian	265(88.3)
	Muslim	35(11.7)
Profession	Medical Doctor	156(52.0)
	Nurse	144(48.0)
Work experience	<1 year	25(8.3)
	1-5 years	215(71.7)
	>5 years	60(20)

**Table 2:** Response of respondents’ to each question that assess their knowledge about PEP

Question	Response	N (%)
Have you heard of PEP	yes	250( 83.3)
	no	50(16.7)
How soon after an exposure should PEP commence	After 72 hours	162(54%0
	Don't know	138(46)
Name the drugs recommended for HIV/PEP	Adequate response ( 3 or more drugs)	86(28.7)
	Inadequate response	96(32)
	Don't know	118(39.3)

**Figure 1:**
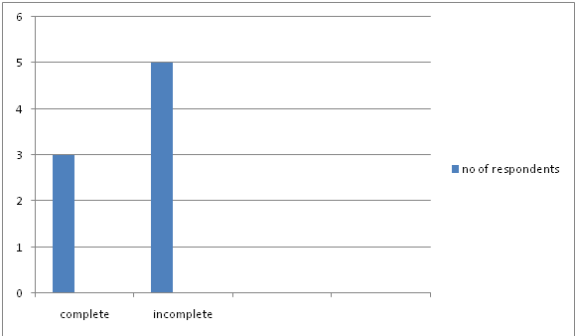
Respondents completion of HIV-PEP after needle stick injury

The effectiveness of PEP for HIV prophylaxis was accepted by the respondents with 219 (73%) respondents accepting to use PEP if the need arises (Table 3). 142 (47.3%) of the respondents have sustained needle stick injury in their medical practice while 158 (52.7%) have never sustained needle stick injury. However, only 123 (87.0%) of the 142 respondents with needle stick injury reported to the appropriate authority while 19 (13.0%) did not report. Reasons for not reporting varied, 7.14% of those who had sustained needle stick injury did not report the incidence because they were unaware of whom to report the incidence to while 34.5% indicated that they did not report because of they were using a new needle (Table 3). Only 8 (6.3%) of the respondents who reported needle stick injuries elected to receive HIV-PEP while 115 (93.7%) did not receive any treatment. Only 3 (37.5%) of the respondents completed the 4 weeks of HIV PEP while 5 (62.5%) did not complete the course (Figure 1).

**Table 3:** Attitude and practise of HIV PEP

Section A: attitude towards PEP	Response	N (%)
Does HIV PEP reduce the likelihood of HIV transmission after occupational exposure	yes	261(87)
	no	80(26.7)
Do you believe PEP works?	yes	219(73)
	no	81(27)
Can HIV be transmitted by needle stick injury?	yes	289(96.3)
	no	11(3.7)
Have you ever sustain needle stick injury during medical practise?	Yes	142(47.3)
	no	158(52.7)
Did you report the needle stick injury?	yes	123(87)
	no	19(13)
**Respondents’ reasons for not reporting needle stick injury**		
**Reason**	**Frequency N = 19**	**%**
New needle	7	36.8
Patient HIV negative	4	21
Unaware of PEP	1	5.3
Injury predates awareness of HIV PEP	3	15.8
fear	2	10.5
Small blood volume	1	5.3
handwash	1	5.3
Have you ever been placed on HIV PEP after needle stick injury?	yes	8(6.3)
	no	115(93.7)

## Discussion

In health care settings there is an increased risk of HIV transmission to HCWs because of occupational exposure to blood borne infection from needle sticks. Evidence suggests that treatment with antiretroviral drugs soon after occupational exposure to HIV decreases the risk of infection. PEP regimes are chosen depending on the type of exposure. Typically regimens are prescribed for a four week period PEP should be started within hours of the potential exposure not days. The sooner PEP is started the better, and it should be started within the first 72 hours after exposure.

HCWs are continuously at risk of transmission of various infections like HIV due to occupational exposure. By training they are taught and continuously practise universal precaution. In this study, majority of the respondents (83.3%) are aware of HIV PEP as expected given their educational background. Despite the high level of awareness, respondents still have an inadequate knowledge about PEP, only 54.0% of respondents knew when to commence PEP following occupational exposure to HIV and less than half of the respondents 46 (15.3%) knew the correct duration for the use of HIV PEP. Among respondents that are aware of PEP, 87.0% are aware that it reduces the transmission of HIV following occupational exposure. These findings are comparable to previous studies done in UK and Spain [9, 10]. A larger percentage of the respondents had a positive attitude about PEP in that they will accept the use of PEP after occupational exposure. This is also not surprising since majority of the respondents are aware that HIV PEP reduces the transmission of HIV following occupational exposure.

There is a high occurrence of needle stick injury among the respondents 47.3% had sustained needle stick injury during their practice. This result is lower than previous study among health workers in the same hospital where 72.9% had a history of needle stick injury [11]. This decrease in occurrence of needle stick injury is due to the provision of a comprehensive universal precaution package and improved disposal of sharps in the hospital. Among those who had suffered needle stick injury, only 41.0% had reported with 21.5% of nurses and 17.3% of doctors reporting the incident. This result is similar to various studies conducted among health worker (especially across Africa), which shows high level of under reporting of needle stick injury among health care workers [3, 12–14].

In this study, of note is the fact that 7.14% of those who had sustained needle stick injury did not report the incidence because they were unaware of whom to report the incidence to while 34.5% indicated that they did not report because of they were using a new needle. This result is comparable to a study done among health care workers in Taiwan where 34% of respondents did not report needle stick injury because the needle near unused [12]. Of great significance is the low level of use of PEP, although the respondents displayed good acceptance of PEP but only 6.3% of respondents who had needle stick injury accepted to use PEP This is not unexpected bearing in mind that majority of the needle stick injury was never reported. Similarly, in a study done by Russi et al, only 13% of the health care workers who reported needle stick injury elected to receive prophylaxis [9].

Also noteworthy is the fact that even out of those that agreed to use PEP, only 4 respondents completed the recommended duration for the use of PEP. The major reason for nonadherence was the unpleasant side effects of the drugs. It is no secret that HIV medication has some unpleasant side effects Because of these side effects the people who have been exposed find it difficult to take their PEP regimen as prescribed and / or complete the four week course. These result in poor adherence and as in the case of HIV infection, poor adherence leads to viral resistance and poor control of HIV. HCWs need to be assured that most symptoms are not serious can be managed. That could make the difference between the PEP being successful or not [1].

## Conclusion

Respondents in this study have good knowledge of HIV PEP and have a positive attitude towards it but there were disparities in their practices of use of the recommended drugs. **Recommendation:** a system that includes written protocols for prompt reporting of occupational exposure, evaluation, counseling, treatment and follow-up should be made available to all health care workers. Health care workers should have access to clinicians who can provide post exposure care during all working hours, including nights and weekends. Antiretroviral agents for PEP should be readily available for timely administration.
